# Effect of Flowering Period on Drone Reproductive Parameters (*Apis mellifera* L.)

**DOI:** 10.3390/insects15090676

**Published:** 2024-09-07

**Authors:** Carlos Castellanos-Zacarías, Álvaro Domínguez-Rebolledo, Henry Loeza-Concha, Jorge Vivas-Rodríguez, Julio Ramón-Ugalde, Juan Baeza-Rodríguez, Roberto Zamora-Bustillos

**Affiliations:** 1 TecNM—Conkal Technological Institute, Technological Avenue S/N, Conkal C.P. 97345, Mexico; carlos.castellanos@itconkal.edu.mx (C.C.-Z.);; 2 Mocochá Experimental Field, National Institute of Forestry, Agricultural, and Livestock Research, Km. 25 Mérida-Motul Old Highway, Mocochá C.P. 97454, Mexico; 3 Postgraduates College, Campeche Campus, Haltunchén-Edzná Highway, Km. 17.5, Sihochac, Champotón C.P. 24450, Mexico

**Keywords:** *Apis mellifera*, flowering scarcity, copulatory apparatus, endophallus, sperm parameters

## Abstract

**Simple Summary:**

In the Yucatán Peninsula, the availability and diversity of plants as food sources for bees vary throughout the year, which impacts dietary composition during the larval phase and compromises the development of their reproductive organs. We evaluated the eversion of the copulatory apparatus, ejaculation, and sperm quality of drones bred during flowering-scarcity (November–December) and flowering-onset periods (January–February). It was observed that more drones had a complete eversion of the copulatory apparatus during the flowering-onset period, with better ejaculation and sperm quality than those bred during the period of flowering scarcity.

**Abstract:**

Insufficient protein intake during the larval phase of drones affects the development of reproductive organs and spermatogenesis. The aim of the study was to evaluate the effect of the flowering-scarcity and flowering-onset periods on the eversion of the copulatory apparatus, ejaculation, and sperm quality of drones (*Apis mellifera* L.). To stimulate the laying of drone eggs during the flowering-scarcity period, 1 L of sugar syrup was supplied weekly to the hives, along with a protein supplement made of 20% pollen and 80% brewer’s yeast. During the periods of flowering scarcity and the onset of flowering, 800 drones were collected (200 per month). At the onset of flowering, 270 drones showed eversion of the copulatory apparatus, of which 162 ejaculated, compared to the flowering-scarcity period, where 112 drones showed eversion of the copulatory apparatus and 39 drones ejaculated. During the period of flowering onset, sperm parameters such as volume, concentration, motility, viability, acrosome integrity, and mitochondrial activity were higher compared to the period of flowering scarcity. In conclusion, we observed a greater number of drones with eversion of the copulatory apparatus and ejaculation, as well as better sperm quality with the drones bred during the period of flowering onset. These differences indicate that the flowering-scarcity period significantly impacts the reproductive parameters of drones.

## 1. Introduction

Beekeeping is of great importance within sustainable agriculture, significantly contributing to economic income, particularly in rural areas [1]. In addition to offering ecological benefits, it enhances crop pollination [2].

The Yucatán Peninsula is among the most important tropical regions for beekeeping [3], and it is one of the leading producers and exporters of high-quality honey, with 95% of its production destined for international markets [4] due to its characteristics of botanical origin and properties of honey [5].

In honey bee (*Apis mellifera*) colonies, drones play an important role in the organization, helping the permanence of the colony through the genetic diversity of sperm, which the queen stores in the spermatheca. The queen, during her mating flight [6,7], mates with multiple drones, which provide enough sperm to fertilize her eggs for the rest of her life [8]. A large number of fertile drones is therefore necessary to ensure optimal mating of the queen [9]. Drone rearing increases in colonies with abundant food resources (flowering period), and to ensure the full growth and development of these drones in their first days of larval life, worker bees nourish them with secretions rich in proteins and amino acids through trophallaxis [10].

Given their larger size, drone larvae have a higher demand for food with a higher protein composition [11] than workers. However, the availability and diversity of food sources fluctuate during the different seasons of the year. In the Yucatán Peninsula, various weather patterns range from semi-arid regions to warm zones and sub-humid climates. In many of these areas, rainfall is mainly concentrated during the summer. A prominent feature of the Yucatán geology is the thin soil layer, whose average thickness is about 20 cm, resting on a calcareous formation of remarkable hardness [12]. In this region, protein resources (pollen) are scarce during certain periods of the year, particularly during the rainy season [13]. Rainfall, temperature, and soil type can lead to drought periods affecting plant phenology, limiting the diversity and availability of nectar and pollen-producing flora [14]. It causes the formation of a small abdomen, which affects the development of the organs inside, including, among them, the size of the testicles and seminal vesicles [15]. It can also influence the ability to evert the copulatory apparatus, preventing ejaculation [16]. Additionally, it has been linked to sperm quality [17], reducing the number of sperm produced and affecting their viability and motility, making it difficult for them to reach and fertilize the ovum.

In recent years, various investigations have been conducted into the reproductive characteristics of drones. The effects of age [18], body size [19], genetic origin [18], temperature [20], nutrition [21], and fresh and frozen semen storage [22], as well as the evaluation of sperm parameters such as volume [23], concentration [18], motility [24], and viability [25] have been studied. However, most of the research in beekeeping on the characteristics and quality of semen in drones has been carried out in countries where climatic conditions, floral diversity, and the availability of raw materials differ from the present study. Currently, there are no research results in regions with a warm, sub-humid tropical climate that explain how the diversity and availability of floral sources that provide nectar and pollen can affect the reproductive success of drones. Therefore, the aim of this study was to evaluate the effect of the flowering-scarcity and flowering-onset periods on the eversion of the copulatory apparatus, ejaculation, and semen quality of drones (*Apis mellifera* L.).

## 2. Materials and Methods

**Place of study:** This work was carried out in the Mocochá Experimental Field at the National Institute of Forestry, Agricultural and Livestock Research (INIFAP). The Experimental Field is located 25 km from the old Mérida–Motul road, located at 21°06′18″ north latitude and 89°27′12″ west longitude at an altitude of 9 m. The predominant climate is sub-humid tropical (Awo), with an average annual temperature of 26.5 °C and an average annual rainfall of 900 mm [12].

**Experimental colonies:** Six experimental double-nucleus colonies with eight frames each, containing newly mated queens from a hybrid genetic line (a cross between African and European bees), were used during both the flowering-scarcity (November–December/rainy season) and the flowering-onset periods (January–February/dry season). The population status of these colonies was stable, with 80% of each frame area covered by adult bees. To stimulate the laying of drone eggs during November and December (a time of year when food resources are scarce in the field), each core was offered 1 L of 50% sugar syrup and 60 g of a protein paste made with 20% pollen and 80% marketed brewer’s yeast, gradually mixed with honey until a smooth and easy-to-handle consistency was achieved [26]. This feeding regimen was provided uniquely 20 days before the month of November on two occasions.

**Experimental design:** A completely randomized design was used, with a total of 400 drones per experimental period (flowering scarcity and onset of flowering), divided into four samples of 100 drones each. The eversion of the copulatory apparatus from each sample was evaluated, and the semen volume was determined from the drones that ejaculated. Subsequently, a pool of the semen obtained was created to evaluate sperm quality.

**Drones capture and handling:** During the period of flowering scarcity, 613 drones emerged, and during the flowering-onset period, 1400 drones emerged. When the drones emerged, they were marked green so they could be monitored at the age of 24 days. Drone collection was carried out using the method described by Neves et al. [27], which consisted of installing a queen excluder at the entrance of the nuclei between 15:00 and 17:00 h. A total of 800 mature drones returning from their flight during the periods of flowering scarcity and the onset of flowering were collected (200 drones per month).

### 2.1. Identification of Floral Resources

**Pollen collection:** During the period of flowering scarcity and the onset of flowering, corbicular pollen samples were collected. The samples were collected using the technique described by Andrada and Tellería [28], which consists of using a pollen trap from 8:30 to 14:30 h. The pollen samples obtained were classified by color to identify their floral origin.

**Classification of pollen granules:** The pollen was subjectively classified using a microscope (LWScientist i40-DNA, LW Scientific, Lawrenceville, GA, USA) at 100× magnification to classify by species according to their color using the identification technique of melliferous flora with ornamental and medicinal potential in the state of Yucatán [29] and by reference to the literature specializing in palynology [30].

### 2.2. Evaluation of Reproductive Parameters

**Figure 1:**
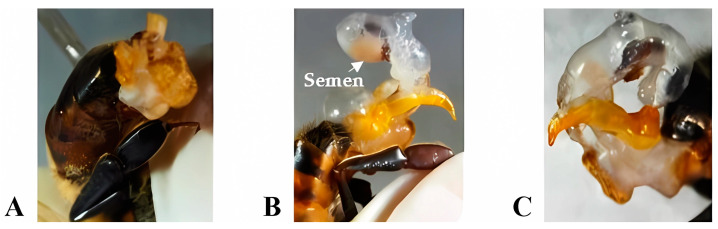
Eversion of the copulatory apparatus: (**A**) Without eversion, (**B**) Eversion with ejaculation, and (**C**) Eversion with absence of semen. (Photos taken by the author).

**Eversion of the copulatory apparatus:** To obtain the percentage of partial and total eversions, the technique described by Collins and Donoghue [31] was applied. It consists of the manual eversion of the endophallus by holding the drone by the head and thorax, ventrodorsally oriented, with the abdomen upwards, between the thumb and index finger, and applying pressure on the abdomen. The result of the technique was classified into A = Without eversion, B = Eversion with ejaculation, and C = Eversion with the absence of semen (Figure 1).

**Volume**: Semen was collected using a large-capacity Harbo Schley^®^ Syringe capillary 50 µL, (Harbo-Schley, Inc., Philadelphia, PA, USA). The sperm was aspirated into the syringe, and immediately, the volume of sperm collected was determined by directly reading the graduation of the syringe.

**Sperm concentration:** The technique described by Taylor et al. [32] was used: 2.5 μL of semen was mixed with 497.5 μL of phosphate-buffered saline (PBS, Thermo Fisher Scientific, Waltham, MA, USA) dilution 1:200. A 9 μL aliquot of the diluted sample was placed on a Neubauer chamber (Hawksley Technology, Lancing, UK, depth 0.1 mm, 1/400 mm^2^) under a phase-contrast microscope (LWScientist i40-DNA, LW Scientific, Lawrenceville, GA, USA) with a 40× objective (UOB UB203i, United Optical Instruments, Tokyo, Japan). The number of sperm in five different chamber areas was counted (each area was equivalent to 25 small squares), and the values were averaged to obtain an accurate estimate. The concentration was then calculated using the following formula:

$$\mathrm{Concentration}\;=\frac{\mathrm{average}\;\mathrm{number}\;\mathrm{of}\;\mathrm{sperm}}{\;\mathrm{volume}\;\mathrm{of}\;\mathrm{the}\;\mathrm{sample}}\;\times \;(\mathrm{dilution})\;$$

**Sperm motility:** This was estimated subjectively using the technique described by Wegener et al. [33]. An amount of 5 µL of fresh semen diluted with 95 µL of PBS was placed on a slide on a Makler^®^ count chamber (Sefi Medical Instruments, Haifa, Israel), preheated to 37 °C, and analyzed using a phase-contrast microscope (LWScientist i40-DNA, LW Scientific, Lawrenceville, GA, USA) with a 10× objective. The percentage of motility was classified on a scale of 1 to 4, where 4 indicates more than 50% of sperm with circular and progressive movements; 3 indicates more than 50% with vibratory, circular, and progressive movements; 2 indicates more than 50% with vibratory movement; 1 indicates less than 50% with vibratory movement; and 0 indicates no movement [34].

**Viability:** This was evaluated using the technique proposed by Tofilsky et al. [35], using the fluorochrome SYBR-14/IP (Live/Dead kit L-7011, Invitrogen, Carlsbad, CA, USA). An amount of 1 µL of SYBR-14 and 1 µL of IP was added to 100 µL of the saline-solution-diluted sperm sample (PBS) and incubated in the dark for 20 min at 37 °C. Subsequently, a 5 µL aliquot of the dyed sample was placed on a slide preheated to 37 °C, and at least 200 spermatozoa were counted with a fluorescence microscope (LWScientist i40-DNA, LW Scientific, Lawrenceville, GA, USA). Those emitting green fluorescence were considered living cells, while dead cells emitted red fluorescence (Figure S1A).

**Acrosome integrity:** This was evaluated using the method described by Fisher et al. [36] using FITC-PSA fluorochrome (100 µg/mL, L0770, Sigma-Aldrich, St. Louis, MO, USA). An amount of 2 µL of fluorochrome was added to 100 µL of the saline-diluted sperm sample (PBS) and incubated in the dark for 20 min at 37 °C. An amount of 5 µL of the stained sample was placed on a slide preheated to 37 °C, and at least 200 sperm were counted with a fluorescence microscope (LWScientist i40-DNA). All cells with green fluorescence emitting from the acrosome were considered damaged, while those with the entire acrosome not emitting fluorescence were considered intact (Figure S1B).

**Mitochondrial activity:** This was estimated according to the technique described by Peña et al. [37], using the fluorochrome JC-1 (153 µM, T-3168, Molecular Probes, Eugene, OR, USA). An amount of 0.5 µL of fluorochrome was added to 100 µL of the saline-diluted sperm sample (PBS) and incubated in the dark for 20 min at 37 °C. A 5 µL aliquot of the stained sample was added to a preheated slide at 37 °C, and at least 200 sperm were counted with a fluorescence microscope (LWScientist i40-DNA). Those that emitted intense green fluorescence from the flagellum were considered active, and those that did not were considered inactive (Figure S1C).

**Data analysis:** Using the PROC FREQ procedure, a chi-square analysis was conducted to assess the independence of the distribution in the eversion of the copulatory apparatus between two periods (flowering scarcity and the onset of flowering). Standardized residuals were examined using the PROC GENMOD procedure to further interpret the results. These residuals help identify which specific combinations of categories significantly influence the overall association detected by the chi-square test.

The Shapiro–Wilk test was performed for the evaluated variables of sperm quality, and the results confirmed that they meet the normality assumption. Each variable’s mean and standard error of the mean (SEM) were computed separately for each period using the PROC MEANS procedure. To statistically compare the sperm quality variables between the two periods, independent-sample *t*-tests (*p* < 0.05) were performed using the PROC TTEST procedure. Statistical data were processed using the Statistical Analysis System (SAS) software 9.4 [38].

## 3. Results

In the Yucatán Peninsula, plant availability and diversity play an important role in sustaining the local bee populations and their apiculture practices. During our study, we observed significant variations in the number of plant species available to bees. Four plant species were identified during the period of flowering scarcity, compared to six species at the onset of the flowering period (Table 1). This fluctuation in floral diversity highlights the seasonal challenges bees face in the region.

The chi-square test revealed a highly significant association (χ^2^ = 121.85, *p* < 0.0001) between the period (flowering scarcity vs. flowering onset) and the eversion of the copulatory apparatus. The residual values indicate significant differences in the stages of copulatory apparatus eversion in drones, with notable variations between the flowering-scarcity and flowering-onset periods (Table S1).

**Figure 2:**
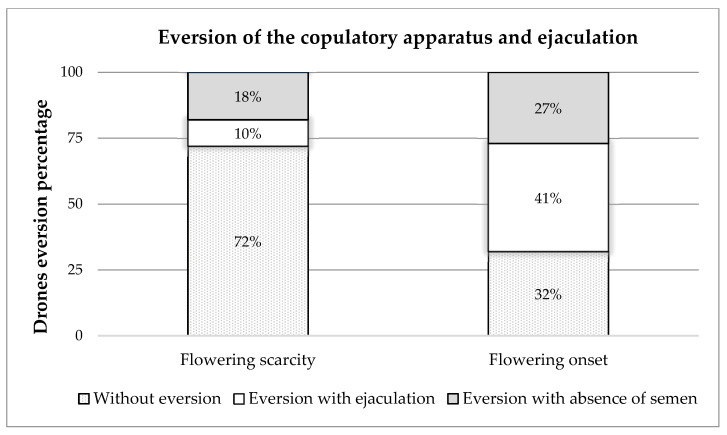
Eversion of the copulatory apparatus and ejaculation of drones bred in the flowering-scarcity and flowering-onset periods.

The results obtained from the eversion of the copulatory apparatus and ejaculation of drones (Figure 1) showed that, during the period of flowering scarcity, of the 400 drones collected, 72% (288 drones) did not evert the endophallus, while 10% (39 drones) showed eversion with ejaculation, and the remaining 18% (73 drones) showed eversion with the absence of semen. At the onset of the flowering period, it was observed that, of the 400 drones collected, 32% (130 drones) did not evert the endophallus, while 41% (162 drones) showed eversion with ejaculation, and 27% (108 drones) showed eversion with the absence of semen (Figure 2).

The sperm quality parameters (volume, concentration, motility, viability, acrosome integrity, and mitochondrial activity) were higher at the onset of flowering compared to the flowering-scarcity period (Table 2).

## 4. Discussion

In this study, it was observed that the diversity and availability of floral resources during the periods of flowering scarcity and the onset of flowering (Table 1) impacted the reproductive parameters of drones (*Apis mellifera* L.). According to Rangel and Fisher [39], drones are susceptible to varying environmental factors and, in particular, the availability of food sources [23] that occur in different seasons [9]. Czekońska et al. [16] observed that pollen limitation during ontogenic development directly affects the reproductive quality of drones. Moreover, Stürup et al. [21] cite that sperm viability is not affected by the lack of protein intake after the drones’ emergence. The above reveals that sperm develops during ontogenetic development (stages and transitions that an organism undergoes during larval development until adulthood). Therefore, the shortage of food sources can affect the development of the copulatory apparatus, compromising the eversion of the endophallus and, thus, ejaculation, which can affect the reproductive behavior of *Apis mellifera carnica* [40], *Apis mellifera ligustica* [23], *Italian hybrid*, and *Buckfast* [18] drones, and other insects such as *Drosophila simulans* [41], *Microplitis rufiventris* [42], *Hermetia illucens* [43], and *Osmia bicornis* [44].

During the period of flowering scarcity, a higher proportion of drones that failed to evert the endophallus was observed compared to the flowering-onset period (72% vs. 32%). This may be because the pollen diet quality influences bee physiology and survival [45]. On the other hand, during the period of flowering onset, a higher proportion of drones undergoing eversion with ejaculation was observed compared to the period of flowering scarcity (41% vs. 10%). These data differ from those published by Czekońska et al. [16], who observed that drones bred with unlimited access to pollen during May and June (flowering period) included a higher percentage that did not evert the endophallus compared to drones that bred with limited access to pollen (13% vs. 8%). However, the rate of drones that ejaculated with semen was higher compared to drones bred with limited access (80% vs. 68%). Kaldún and Otti [46] observed that in common male bedbugs (*Cimex lectularius*), restricting their food led to reduced production of sperm and seminal fluid compared to fully fed males. Moreover, Czekońska and Chuda-Mickiewicz reported that the ability of drones to evert the endophallus and ejaculate is related to anatomical changes or the absence of semen in the seminal vesicles, probably due to the delayed development of the mucous glands [40].

During the flowering-onset period, a significant increase in semen volume (0.9 μL vs. 0.5 μL), an increase in sperm concentration (5.6 × 10^6^ vs. 4.1 × 10^6^), motility (80% vs. 70%), viability potential (90% vs. 75%), acrosome integrity (83% vs. 70%), and mitochondrial activity (75% vs. 60%) were observed, compared to the period of flowering scarcity. A higher semen volume is associated with a lower degree of polyandry in insects, as observed in the species *Apis cerana* [47]. In the honey bee *Apis mellifera scutellata*, a higher volume of semen contains more seminal fluid, which helps to improve metabolic activity in the sperm cells, giving them energy for their mobility [48]. Mobility (motility) is fundamental for sperm migration through the lateral and central oviducts to reach the ovum [49]. Mitochondrial activity drives the movement of sperm to the egg through oxidative phosphorylation, a fundamental process to produce ATP (energy) [50]. In our study, fluorochrome JC-1 stained the flagellum of active mitochondria with intense green fluorescence (Figure S1C), similar to what was observed in rat sperm mitochondria using the same JC-1 fluorochrome [51]. Moreover, Buffone [52] explains that the sperm acrosome contains enzymes, such as hydrolase, which are necessary to dissolve the egg’s outer layer during fertilization. In the hamster, the acrosin enzyme is essential for sperm penetration into the pellucid zone [53].

The data obtained in this research are different from those of Morais et al. [54], who observed improvements in drone sperm concentration, motility, and viability during the rainy season (March–June) compared to the dry season (October–December). This discrepancy may be influenced by the specific environmental conditions of each study, as variability in the quality and availability of floral resources between different seasons and regions could impact drone physiology differently. Similarly, Rhodes et al. [9] found that drones bred in the spring (during flowering) produced higher semen volumes than those bred in the summer and autumn (flowering scarcity). It is important to consider that not all floral resources are equal in terms of nutritional quality and pollen accessibility [55,56], which could explain the observed differences in semen volumes. Variations in floral biodiversity and pollen quality across different locations and seasons may influence the ability of drones to fully develop their reproductive systems, potentially affecting sperm production, viability, and overall reproductive success. However, Czekońska et al. [16] noted that sperm concentration and viability do not differ in drones when they are bred with limited versus unlimited access to pollen. It could be argued that the variations in results are partly due to how pollen limitation is defined and measured in each investigation. In this study, the scarcity of floral resources significantly impacted the reproductive parameters of drones, indicating that both the severity and duration of this limitation are crucial factors to consider. Rousseau and Giovenazzo [23] noted that the sperm concentration in drones remains constant when they receive an energy- and protein-rich diet in the spring. However, the volume of semen produced and sperm viability show improvements with this diet. It is reasonable to consider that differences in the quality and type of supplementation, as well as the initial nutritional state of the colonies, could affect the results. On the other hand, Fisher et al. [36] noted that forage availability could play a more significant role in drone fertility rather than the genetic diversity between colonies.

It is worth mentioning that other factors can affect the reproductive quality of drones. Koeniger et al. [57] stated that sperm concentration varies depending on the drones’ genetics. The same authors also mentioned that *Apis dorsata* drones have less sperm concentration than the *Apis mellifera* species. Fisher and Rangel [58] commented that exposure to insecticides present in beeswax decreases sperm viability and, thus, their ability to fertilize the queen. Moreover, Bratu et al. [59] point out that drones with higher body weight produce semen with higher sperm concentrations compared to drones with lower body weight. However, Metz and Tarpy [60] mentioned that drones at 20 days of age reach a sperm concentration of 7.39 × 10^6^ sperm and that this decreases after 30 days of age. Also, Czekońska et al. [61] commented that the semen volume decreases and the sperm viability increases as the drone ages.

These findings underscore the profound interconnectedness between environmental resources and biological processes. The variability in the availability and quality of floral resources during drone larval development highlights how external factors influence drone physiology and reproduction success.

## 5. Conclusions

The drones collected at the onset of the flowering period present greater eversion of the copulatory apparatus, eversion with ejaculation, and better sperm quality compared to the drones obtained in the flowering-scarcity period. These differences indicate that the flowering-scarcity period significantly impacts the reproductive parameters of drones, which could be related to environmental factors such as excessive rainfall or biological factors such as the presence of parasites, which vary between these periods. To mitigate these negative effects, it is essential to implement management practices that ensure the availability of floral resources throughout the year, such as using plants with extended flowering periods or providing supplementary feeding to colonies during critical periods. However, it is crucial to conduct more detailed studies investigating how the diet provided by nurse bees during drone larval development influences sperm quality at the metabolomic and proteomic levels and how these epigenetic effects might alter the observed outcomes.

## Figures and Tables

**Table 1:** Floral species were identified as feeding sources for bee colonies during the flowering-scarcity and flowering-onset periods.

Family	Period/Species	
	Flowering Scarcity	Flowering Onset
Asteraceae		*Sclerocarpus divaricatus*
Convolvulaceae	*Ipomoea crinicalyx*	*Ipomoea hederifolia*
	*Jacquemontia pentantha*	*Jacquemontia nodiflora*
		*Jacquemontia pentanthos*
Leguminosae	*Mimosa bahamensis*	*Leucaena leucocephala*
		*Acaciella angustissima*
Verbenaceae	*Lantana camara*	

**Table 2:** Spermatic parameters of drones (*Apis mellifera* L.) bred in flowering-scarcity and flowering-onset periods (Mean ± SEM).

Sperm Parameters	Period	
	Flowering Scarcity	Flowering Onset
Number of drones	39	162
Volume (μL)	0.5 ± 0.3 ^b^	0.9 ± 0.79 ^a^
Concentration (×10^6^)	4.1 ± 1.47 ^b^	5.6 ± 1.01 ^a^
Motility (scale 1–4)	70 (e3) ± 4.23 ^b^	81 (e4) ± 4.85 ^a^
Viability (%)	75 ± 3.97 ^b^	90 ± 2.40 ^a^
Acrosome integrity (%)	70 ± 3.21 ^b^	83 ± 3.75 ^a^
Mitochondrial activity (%)	60 ± 5.30 ^b^	75 ± 4.10 ^a^

^(a,b)^ Different superscripts in the same row express significant differences between periods. (e3) = vibratory, circular, and progressive movement. (e4) = circular and progressive movement. Level of significance (*p* < 0.05).

## Data Availability

The data are available upon request from the corresponding authors: baeza.juanjose@inifap.gob.mx and roberto.zb@conkal.tecnm.mx.

## Supplementary Materials

The following supporting information can be downloaded at https://www.mdpi.com/article/10.3390/insects15090676/s1, Figure S1: Microscopic images: [A] (a) live sperm, (b) dead sperm; [B] (a) inactive mitochondria, (b) active mitochondria; and [C] (a) damaged acrosome, (b) intact acrosome. Table S1: Chi-square analysis of the eversion of the copulatory apparatus in drones during the flowering scarcity and flowering-onset periods.
