# Supplementary material for: Effect of Flowering Period on Drone Reproductive Parameters (Apis mellifera L.)
Source: Insects. 2024 Sep 7;15(9):676. doi: 10.3390/insects15090676 (PMC11432442; doi:10.3390/insects15090676)
Supplement: Supplementary file 1 [file insects-15-00676-s001.zip › Table S1. Chi-square analysis of the eversion of the copulatory apparatus in drones during flowering scarcity and onset flowering period..pdf]

**Table S1:** Chi-square analysis of the eversion of the copulatory apparatus in drones during flowering scarcity and onset flowering period.

| Eversion of the copulatory apparatus | Period | Frequency | Expected | Standardized residuals | Chi-square | Pr > ChiSq |
|---|---|---|---|---|---|---|
| Without eversion | F. Scarcity | 288 | 209 | 5.46454~ | 121.85 | <.0001 |
| Without eversion | O. Flowering | 130 | 209 | -5.46454~ | | |
| E. with semen | F. Scarcity | 39 | 100.5 | -6.13468¤ | | |
| E. with semen | O. Flowering | 162 | 100.5 | 6.13468¤ | | |
| E. with absence of semen | F. Scarcity | 73 | 90.5 | -1.83956 | | |
| E. with absence of semen | O. Flowering | 108 | 90.5 | 1.83956 | | |

E= ejaculation, F= flowering, O= onset. (~,¤) Equal symbols indicate significant differences between the periods of 'flowering scarcity' and 'onset flowering' in the behaviors 'Without eversion' and 'Ejaculation with semen' as demonstrated by the standardized residuals ($p < 0.0001$). The behavior 'Ejaculation with absence of semen' showed variations in the residuals that, although less pronounced, did not significant difference ($p > 0.0514$).
