# Supplementary figures and images for: Effect of Flowering Period on Drone Reproductive Parameters (Apis mellifera L.)
Source: Insects. 2024 Sep 7;15(9):676. doi: 10.3390/insects15090676 (PMC11432442; doi:10.3390/insects15090676)

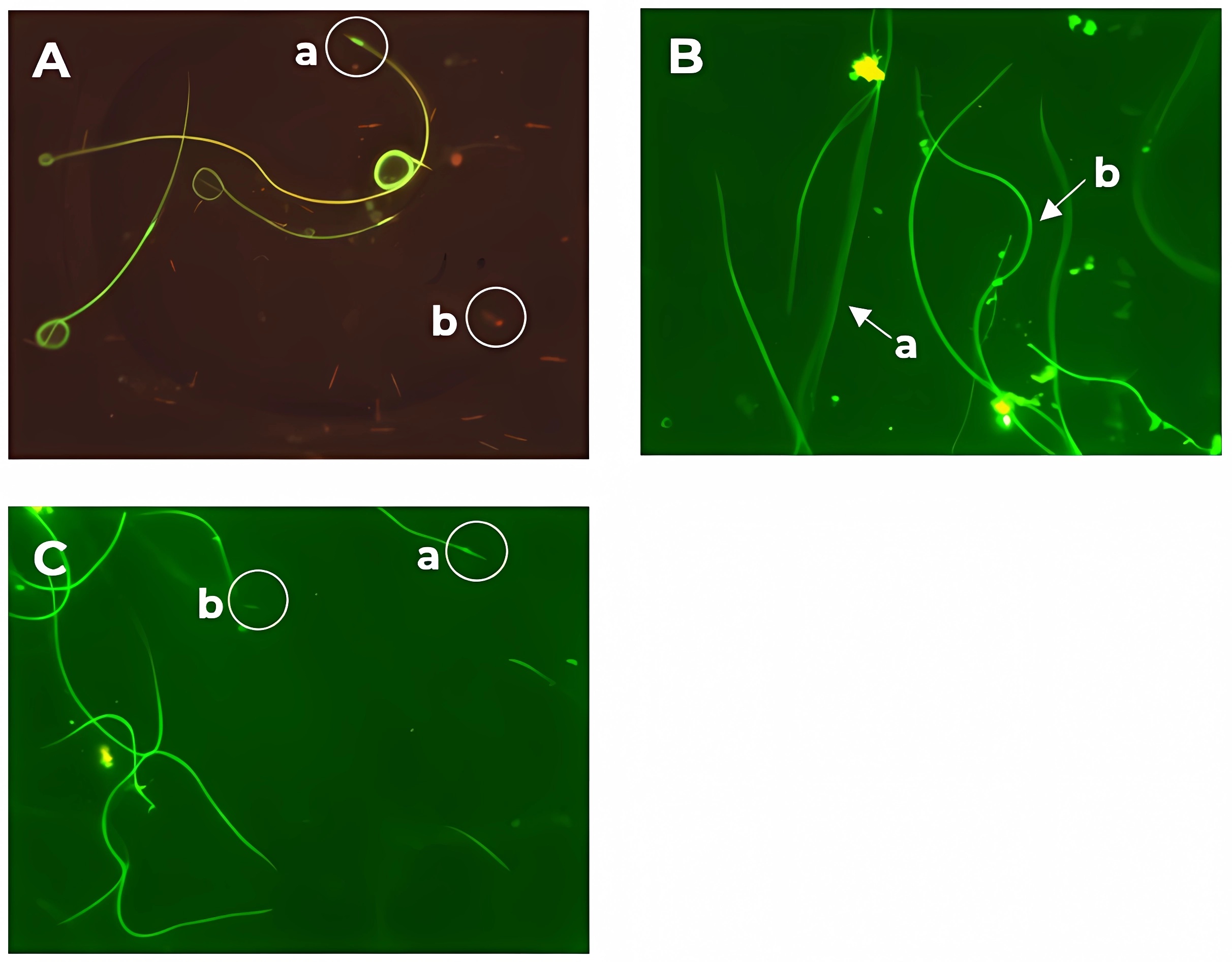

Supplement: Supplementary file 1 [file insects-15-00676-s001.zip › Figure S1. Microscopic Images [A] (a) Live spermatozoa, (b) Dead spermatozoa; [B] (a) Inactive mitochondria, (b) Active mitochondria; [C] (a) Damaged acrosome, (b) Intact acrosome..jpg]
